# The development of transcatheter aortic valve replacement (TAVR)

**DOI:** 10.21542/gcsp.2016.32

**Published:** 2016-12-30

**Authors:** Alain Cribier

**Affiliations:** Dept. of Cardiology, University of Rouen, France

## Introduction

The development of transcatheter aortic valve replacement (TAVR) can certainly be considered one of the most fascinating examples of successful translational research in medicine. Thanks to an outstanding partnership between multidisciplinary clinicians and engineers, we could move from concept to bench, bench to bedside, bedside to clinical feasibility trials, then on to larger clinical registries and evidence based trials, leading ultimately to a breakthrough technology with durable impact on the pattern of medical practice.

This disruptive technology evoked scepticism and criticism in the beginning, but thanks to innumerable clinical trials and evidence based investigations, it is now widely accepted by the medical community and its acceptance is continuing to grow. In the last fourteen years, TAVR has been performed in around 300,000 patients in 65 countries and adoption is increasing by 40% year on year.

The field of TAVR is rapidly evolving, with major refinements in technology, procedural techniques, patient selection and biomedical engineering. With the development of better devices, new approaches and new implantation strategies, TAVI has become much simpler and safer. The indications were initially limited to elderly aortic stenosis patients with multiple co-morbidities. The same are now cautiously and appropriately growing to include a broader population of patients with lower surgical risk, degenerated surgical bioprosthesis, and even patients with other valvular diseases such as pure aortic or even mitral insufficiency. There are few examples of clinical fields in medicine that match the rapid and careful evolution of TAVI.

## Background

**Figure 1.**
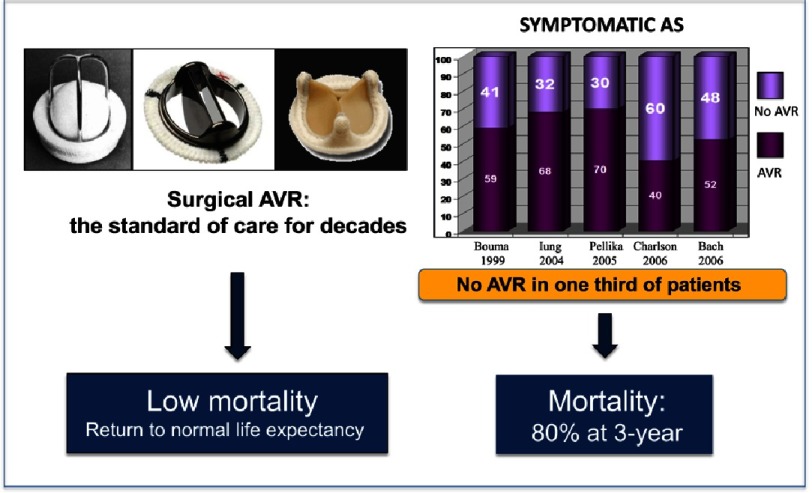
Rational for developing interventional technologies for severe AS: An Unmet Clinical Need.

Calcific aortic stenosis (AS) is the most frequently acquired valvular heart disease in developed countries, and its prevalence increases with an ageing population.^1^ The natural history of symptomatic aortic stenosis carries a poor prognosis^2^ with a survival rate of 60% and 32% at one and five years respectively.^3^ The only effective treatment for decades was surgical aortic valve replacement (SAVR) with remarkable results in ideal candidates, but which required invasive heart surgery with extracorporeal circulation. Operative mortality of SAVR is low, <5%^4^ and alleviation of symptoms and a return to normal life expectancy are observed. However, the operative risks, including post-operative complications and mortality, significantly increase in very old patients and/or in the presence of associated cardiac or non-cardiac comorbidities.^5,6^ These factors are considered one of the main reason for which at least one-third of patients with symptomatic AS are not referred for SAVR^7^ (Fig. 1).

In the 1980s, age of over 75 years was considered a contraindication of SAVR, and this stimulated our group to develop a less invasive therapy, balloon aortic valvuloplasty (BAV), consisting of enlarging the calcified native valve with a balloon catheter using standard catheterization techniques.^8^ This technology was adopted with enthusiasm by the medical community, as highlighted by the thousands of patients included in broad European and US registries and the 1,300 indexed articles dedicated to the procedure.

However, the enthusiasm progressively declined following the recognition of important limitations, headed by early valve restenosis. BAV appeared to provide only temporary relief of symptoms with a modest survival benefit^9,10^, its role remaining controversial in US guidelines.^11^ Interest in BAV resurged with the development of TAVR and its frequent integration in the procedure. BAV is also used today as a palliative option in patients with contra-indication to TAVR or SAVR, as a bridge to those procedures in severely depressed left ventricular function, or when urgent non-cardiac surgery is indicated. Even though age is no longer considered a surgical contraindication, large numbers of severe AS patients are not offered valve replacement in Europe or the United States.^12,13^

## From balloon valvuloplasty to the concept of percutaneous aortic valve

**Figure 2.**
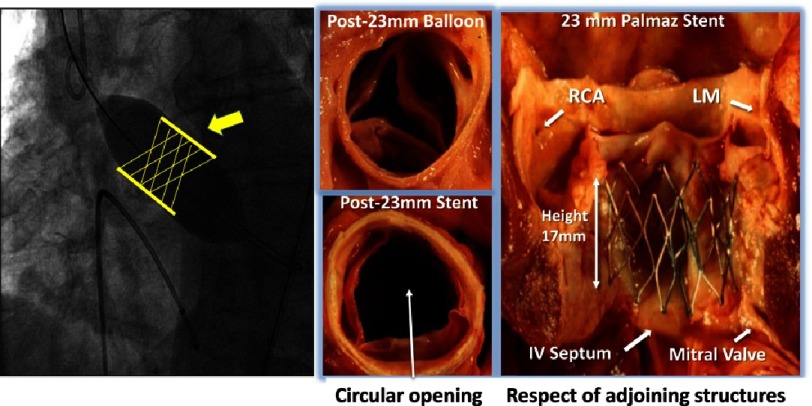
Birth of the idea of “stented-valve” in AS. *Left panel*: A stent crimped over a high-pressure valvuloplasty balloon might keep the valve open and prevent restenosis. A valve structure should be added within the stent. *Right panel*: Validation of the concept of intra-valvular stenting and optimal height of the frame to respect adjoining structures.

For those of us who had been pioneering BAV, addressing the issue of post-BAV valvular restenosis became an obsession in the early 1990s. Placing a balloon expandable stent frame containing a valvular structure (stented-valve) within the calcified native valve appeared a possible option (Fig. 2). The project had the advantage of requiring similar approaches and techniques to those used for BAV. Among several visions of endovascular valve implantation, with initial animal investigation performed by Davies,^14^ H. R. Andersen’s project was the most elaborated. In 1992 he developed and patented^15^ a hand-made “stented valve” for the treatment of various cardiovascular diseases, but the project remained at the experimental stage. In 2000, Bonhoeffer first used a stented-valve in a human, a bovine jugular vein in a metallic stent to treat degenerative ventriculo-pulmonary conduits in children.^16^

Our goal to implant a stented valve in calcific AS, on the beating heart, was very original but posed specific, difficult and at first sight insurmountable issues. These issues came from the calcified nature of the diseased native valve, and the immediate proximity of essential anatomical structures: coronary ostia, mitral valve, and interventricular septum (seat of the conduction system).

## Validation of intra-aortic valve stenting and feedback of experts

To validate the concept of intravalvular stenting in aortic stenosis, an autopsy study was conducted in Rouen in 1994 on 12 cases of calcific AS (Fig. 2). The study demonstrated that a balloon-expandable peripheral artery stent of 23 mm in diameter (Palmaz stent) was able to maintain a circular opening in all calcified aortic valves. The study also made it possible to establish the optimal dimensions of the stent height, avoiding any contact with the neighboring structures. Furthermore, the stent required a high traction force to be dislodged from the annulus, thus lowering the potential risk of device embolization.

**Figure 3.**
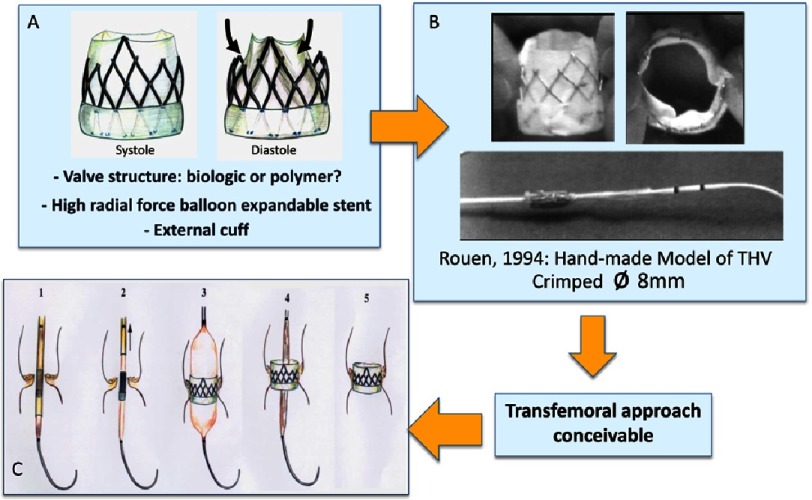
1994: Drawings and model prefiguring a balloon expandable transcatheter bioprosthesis. A: specific stent frame design allowing to attach a tricuspid valvular structure. Partial external coverage would limit the risk of aortic regurgitation through the struts. B: Hand made model of stented-valve before and after crimping over a balloon catheter (external diameter: 8 mm). C: Drawing of the different phases of transcatheter aortic valve implantation.

This study was a fundamental milestone and validated the concept of aortic valvular stenting in a model of human calcific AS. At that stage, the type of valve prosthesis and its physical properties were still limited to drawings, however they were still used to file a European patent (Fig. 3).

Getting biomedical companies interested in this concept was a total failure with unanimously unfavorable opinions from all experts with regard to the design of the prosthesis, the potential risks of the procedure and the medical indication itself. Major clinical issues were constantly brought up: coronary occlusion, mitral valve injury, stroke, aortic regurgitation, prosthesis migration, permanent auriculo-ventricular block, bleeding, endocarditis, and non-lasting results. The project was looking like the “most stupid ever proposed”.

## From concept to prototypes: pre-clinical evaluation

### Creation of the start-up: Percutaneous Valve Technologies

**Figure 4.**
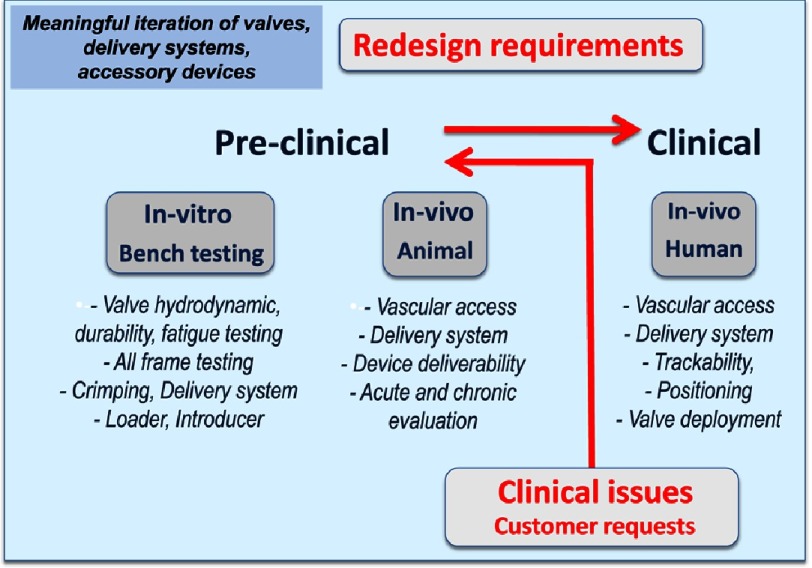
The translational pathway of transcatheter aortic valve replacement: driving for superior outcomes.

To accomplish this venture, a start-up company, ‘Percutaneous Valve Technologies’ (PVT, NJ, USA) was finally formed in 1999 (Alain Cribier, MD, Martin Leon, MD, Stan Rabinovich and Stanton Rowe, PhDs). A development and first investment partner was found in Israel (ARAN, R&D, Ltd, Caesarea) a small biomedical company with great engineers which became our long-lasting partner in this venture. This was the start of a strong, durable and successful collaboration between engineers and clinicians. The translational pathway to TAVR, set by PVT and ARAN, would remain unchanged in the future for all companies working on the development of such a procedure (Fig. 4).

#### Preclinical engineering output: From concept to finalized prototype

Indications given to the engineers for the development of a transcatheter heart valve (THV) were particularly challenging. They had to integrate many innovative technologies: a balloon-expandable stent, a high-pressure balloon for stent expansion, a valvular structure and a delivery system. According to the “philosophy” of the THV, they had to create a prosthesis made of a highly resistant frame containing a valve structure, able to be homogeneously compressed to 7-9 mm over a high pressure balloon (trans-femoral artery insertion) and expanded to a diameter of 23 mm by balloon inflation, without damaging the frame and leaflets. Selection of the valve material, conceiving its attachment to the frame, and the valve design to provide sufficient strength, low profile and durability were other issues. The question of how to deliver the valve accurately, within the calcified valve, on the beating heart, would come later.

Many different valve configurations were investigated. Valve design was dependent on:

**Figure 5.**
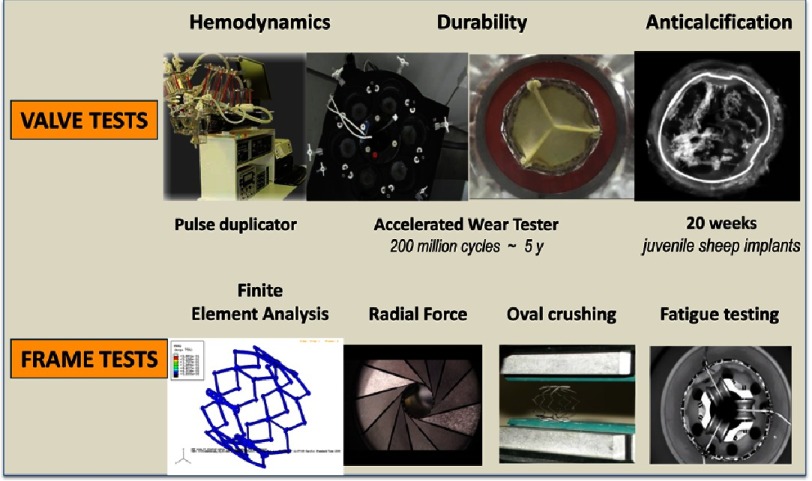
New testing equipment designed by PVT for the evaluation of valve structure and frame.

1. Frame material and design (profile, dimensions, skirt, crimping process and expansion, fatigue, resistance).
2. Leaflets design (material, attachments, cooptation, stress distribution, leak, hemodynamics, fatigue and durability, calcification).
3. Loading and delivery catheter system.

Each of these elements required specific work on design-geometry, material selection, manufacturing and processing. Geometry optimization used the Finite Element Analysis (FEA) method. The goal was to maintain the durability constraints while reducing the crimping profile. For laboratory testing (Fig. 5), the company had to design its own equipment for a new technology: crimping tools, pulse duplicators, accelerated wear and durability testers, various frame testers, hydrodynamic testers, and a leaflet calcification tester.

**Figure 6.**
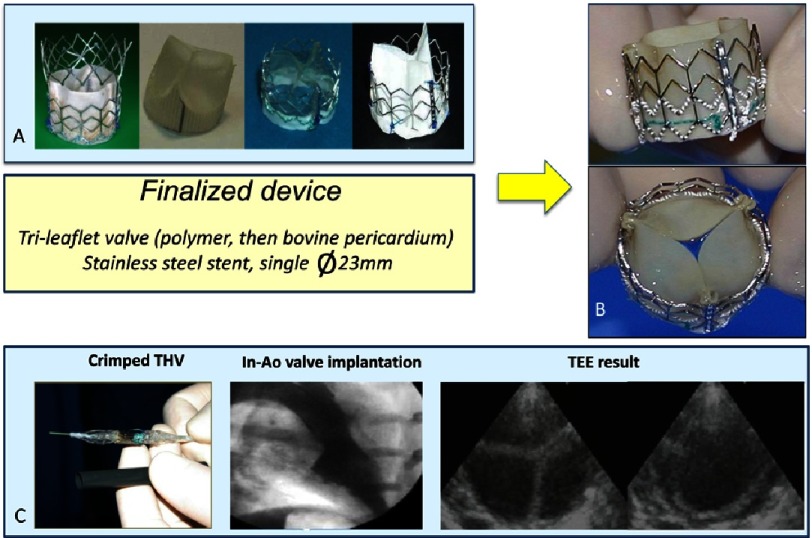
A: Various prototypes and finalized device (B) created by PVT. C: Crimped device over a 23 mm Numed balloon catheter, and 24F introducer for implantation in the sheep model. Angiographic evaluation post-implantation within the native aortic valve, and transesophageal echocardiography evaluation of valvular function.

The first “finalized” device (Fig. 6) consisted of a stainless steel stent, 23 mm in diameter, 17 mm in height, containing a tri-leaflet valve initially made of polyurethane (later changed to a bovine pericardium valve), which had been proven for more than 25 years in surgical bioprosthesis to have excellent properties. The device was compatible with a 24F (8 mm) introducer sheath.

## From prototypes to animal model

In the year 2000, we started animal experimentation on the sheep model (Fig. 5). Over 100 THV implantations at various cardiac sites (pulmonary artery, aorta, aortic valve) were performed by myself and my collaborator, Helene Eltchaninoff. In spite of the clear limitations of this animal model, the experimentation contributed to the optimization of bioprosthesis, delivery systems, and implantation techniques, guidewires and procedural aspects (assessment of annulus size, accuracy of valve positioning, optimal X-ray projection, technique of valve delivery, methods of cardiac standstill, evaluation of results by angiography and echocardiography, anticoagulant strategy).

Chronic (5-month) evaluation in the systemic circulation was obtained using an original method of THV implantation in the descending aorta.^17^ This was mandatory before being committed to FIM trial as post-durability testing and as a test of biocompatibility. The persistence of an excellent valve function and the integrity of the THV on pathological examination were thus demonstrated.

## From bench to bedside

**Figure 7.**
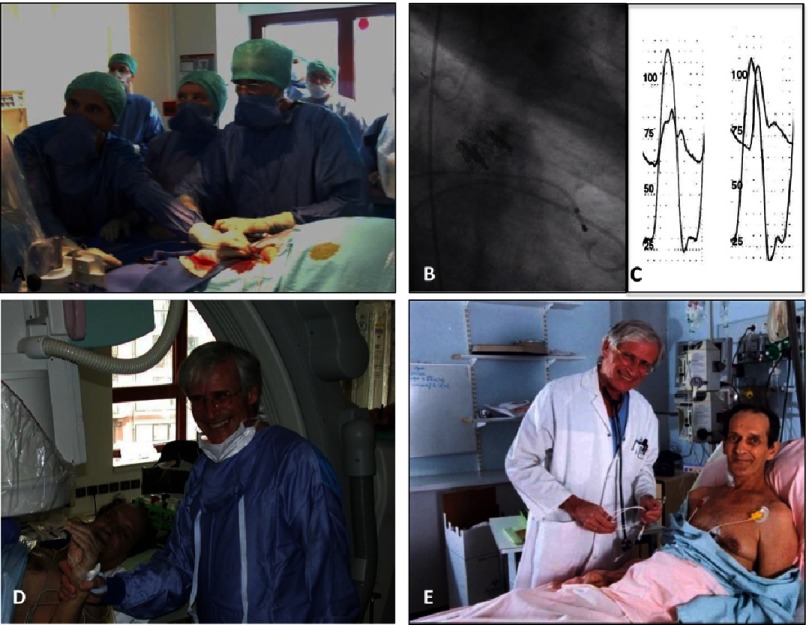
First-in-Man implantation (Rouen, April 16th, 2002). A—The complex antegrade transseptal route used for TAVR. B—View of the transcatheter valve in place within the native calcified valve and hemodynamic result (no gradient). C—The patient immediately after valve implantation and D, 8 days later.

On 16th April 2002, we performed the first-in-human TAVR (Fig. 7) on a 57-year-old patient with severe AS who presented in cardiogenic shock with major left-ventricular dysfunction (ejection fraction 12%) with multiple comorbidities contraindicating SAVR.^18^ After failed emergent BAV, TAVI appeared to be the last-resort option for this young patient. The indication was particularly challenging in this patient, who also had subacute leg ischaemia related to an aorto-femoral bypass occlusion and severe contralateral atherosclerosis preventing the use of the planned transfemoral retrograde access. The procedure was successfully performed using a challenging approach, the antegrade transseptal approach via the femoral vein. The THV could be accurately deployed in the middle of the valvular calcification. After deployment, the patient’s hemodynamic and echocardiographic status improved remarkably.

From a single case, the feasibility of THV implantation on the beating heart using transcatheter techniques was confirmed. There was no coronary occlusion, no mitral dysfunction, no atrio-ventricular block and only a mild paravalvular aortic regurgitation, thus translating well our 1994 post-mortem study. The patient unfortunately died four months after the procedure, due to complications unrelated to TAVR (leg amputation consecutive to his pre-hospitalization leg ischemia). This first-in-man case confirmed the feasibility of implanting a THV in a human on the beating heart using transcatheter techniques, with perfect subcoronary position and no interference with the surrounding structures. In that, it can be considered an important milestone in interventional cardiology. The international reaction to this spectacular case defied imagination.

Two successive feasibility trials on a total of 38 patients^19–21^ restricted to compassionate use (imminent death) were thereafter initiated in our center. These studies confirmed the feasibility of TAVI (80% procedural success) using the transseptal approach and the lasting haemodynamic and functional improvement after implantation. However, a high (25%) incidence of > grade 2 paravalvular regurgitation was noted, indicating an insufficient coverage of the annulus in a number of patients and the need to develop larger size bioprosthesis ( >23 mm).

As expected, several of these critically ill patients died of their comorbidities within weeks or months but, amazingly, some survived beyond 2–5 years and even as long as 6.5 years in our most striking case, without any prosthesis dysfunction. Protocol extension to other centers in Europe, USA and Canada was started but demonstrated a significant degree of technical complexity and adverse outcomes associated with the antegrade delivery. In our series, TAVR was also attempted in 7 patients using the initially-planned, and technically simpler, transfemoral retrograde approach. The procedure was carried out successfully in 4 patients in spite of the lack of any specific delivery system adapted to this route. Obviously, further expansion of TAVR required technical improvements, procedure simplification, more friendly approaches and larger valve sizes.

**Figure 8.**
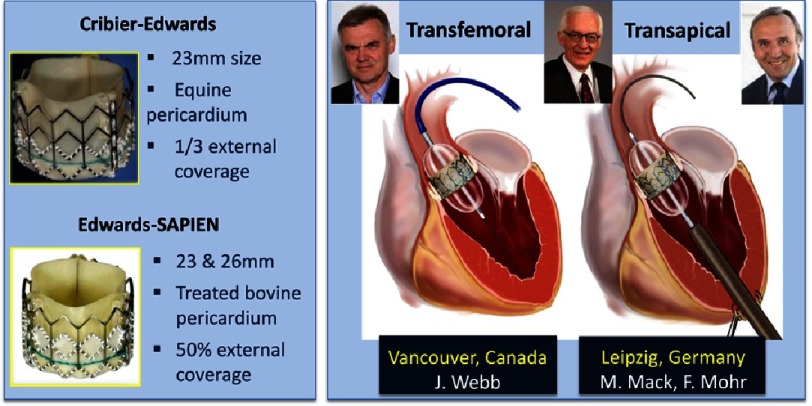
Edwards Lifesciences input after acquisition of Percutaneous Valve Technologies (2004): development of the SAPIEN valve and of new approaches for TAVR: transfemoral retrograde and transapical antegrade.

## From bedside to feasibility trials

When Edwards Lifesciences Corporation (Irvine, CA, USA) acquired PVT in 2004, TAVR entered a new era. The prosthesis underwent several iterations and an easier delivery system and new approaches were developed (Fig. 8).

The Edwards-SAPIEN (originally Cribier-Edwards) valve prosthesis became available in two diameters: 23 mm and 26 mm. This model of bioprosthesis consisted of a tri-leaflet bovine pericardium valve pretreated to decrease calcification, mounted within a stainless steel stent externally covered by a longer pet cuff (50% versus 33% of the frame height).

A specific delivery system was conceived for facilitating the retrograde transfemoral approach, the deflectable RetroFlex catheter, evaluated by Webb et al. in Vancouver, Canada.^22^ Simultaneously, a new approach was developed, the minimally invasive transapical approach using the Ascendra delivery system, evaluated by Walther et al. in Leipzig, Germany.^23^ The onset of these two approaches made TAVR available to the vast majority of patients, regardless of the suitability of the femoral access. Our team in Rouen was included in the setting of several European feasibility studies (REVIVE, PARTNER Europe, TRAVERSE) including hundreds of patients. The satisfactory results of these trials, despite specific complications with the two approaches, led to a fast expansion and acknowledgement (in particular by cardiac surgeons) of TAVR.

In 2004, a concurrent THV, the CoreValve (later commercialized by Medtronic, Irvine, CA, USA), an auto-expandable nitinol frame containing a porcine pericardial valve, was launched and evaluated in feasibility studies.^24^ This device could be inserted via a transfemoral approach through smaller sheath sizes (21F then 18F) than those required for Edwards devices (22F and 24F). As an alternative to the femoral delivery, the subclavian access was proposed with the CoreValve. The Conformité Européenne (CE) mark was obtained for both models of transcatheter valves in 2007.

## From feasibility trials to larger clinical registries and evidence-based trials

Thereafter, acceptance and expansion of TAVR was amazing, with an annual 40% increase in the number of procedures. In line with the recommendations of the European Societies of Cardiology (ESC) and Cardiothoracic Surgery (EACTS),^25^ thousands of inoperable or high-risk elderly patients were enrolled in post-marketing national (France, Germany, Italy, UK, Canada etc.) and international registries with the two models of THV.

These registries included:

• Single valve evaluation as in the SAPIEN Aortic Bioprosthesis European Outcome (SOURCE) registry,^26^ which has enrolled 1,123 patients since 2007 receiving transfemoral or transapical TAVR.
• The Evaluation of the Medtronic CoreValve System in a “Real-World” (ADVANCE) Registry, presented at the EuroPCR meeting in Paris, in May 2013, including 1,015 patients enrolled at 44 centers.
• Two valve evaluations: the French Aortic National CoreValve and Edwards (FRANCE) registry,^27^ followed by the FRANCE 2 registry,^28^ reporting the French experience on a series of 3,500 patients, making it the largest exhaustive overview of TAVR in the real life.

These registries contributed to a better appraisal of patient screening, technical modalities, prevention, and management of complications. The procedural success rate increased to over 95%, and with advanced technologies, immediate and long-term results kept improving. The hemodynamic results were shown to compare favorably with surgical valve replacement in similarly ill patients. The results of TAVR became more predictable and the mortality rate decreased to 10% at 1 month and 20% at 1 year, as in the SOURCE registry,^26^ after transfemoral implantation. A dramatic and long lasting improvement in the quality of life^29^ was observed in all registries, and was further confirmed in the pivotal PARTNER trial.

The first evidence-based evaluation of TAVR was obtained with the Edwards SAPIEN valve in the multicenter pivotal randomized trial “Placement of Aortic Transcatheter Valves” (PARTNER) in the USA. From 2007, 1,056 high surgical risk patients were enrolled in 26 centers in USA. Patients were divided into two cohorts, a non-surgical arm (Cohort B) in which TAVR was compared with medical therapy (including BAV); and a surgical arm (Cohort A) in which transfemoral or transapical TAVR was compared to traditional SAVR.

Briefly, the results confirmed the high superiority of TAVR over medical treatment in non-operable patients with an absolute increase in survival of 20% at 1 year, and the non-inferiority of TAVR versus SAVR in high-risk operable patients in terms of all-cause mortality and repeat hospitalization at 1 year, with equal improvement of quality of life^30,31^.

Similar results were observed at 2, 3 and 5 years.^32–34^ In view of these results, TAVR was approved by the Food and Drug Administration (FDA) in these indications in 2011 and 2012 respectively. The pivotal CoreValve high-risk trial also randomized TAVR vs SAVR in symptomatic high-risk patients with severe AS, with a primary end point of all-cause mortality at 1 year. This trial was the first and so far the only randomized trial to ever show superiority for TAVR vs SAVR (14.2 vs 19.1% respectively), results confirmed at 2 years.^35,36^ In these trials, the similarity or superiority of transcatheter over surgical valves on hemodynamic flow parameters, but the superiority of surgical valve on paravalvular leak and the need for a permanent pacemaker were observed.

**Figure 9.**
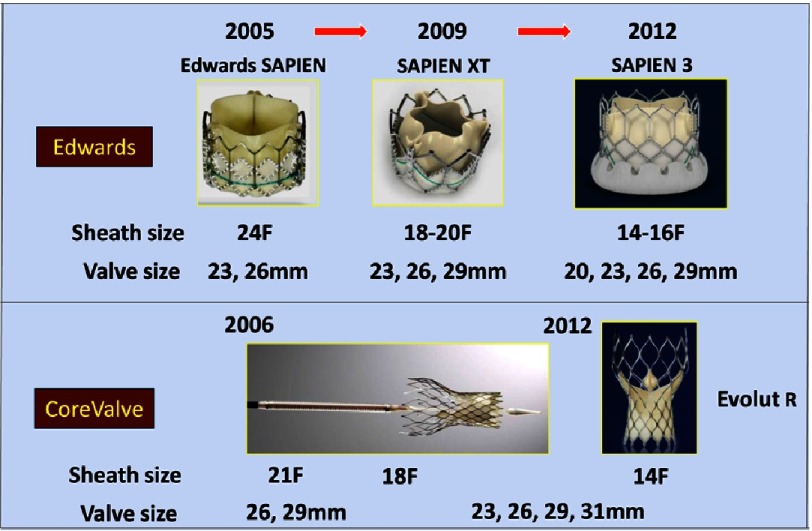
Advanced valve and delivery systems have changed the world of TAVI overtime. Several generations of Edwards and Medtronic CoreValve led to decreased crimped sizes and launch additional valve sizes for a better coverage of the aortic annulus.

**Figure 10.**
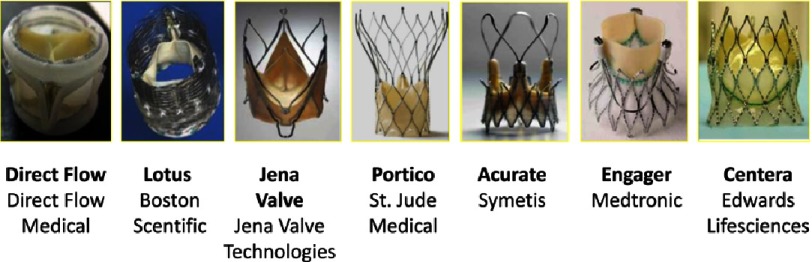
New models of bioprosthesis approved in Europe.

## Solving the problems: the essential role of translational research

After several years of experience, the task of the engineers was to improve both the technological aspects of TAVR, while reducing the complications. Severe vascular complications (3–16%), stroke (2–7%), paravalvular aortic regurgitation (AR: 5% > grade 2), and complete heart block requiring pacemaker (PM: Edwards 3–12%, CoreValve 16–35%) were the leading complications.^37^

Improvements were achieved by creating new models of bioprosthesis and delivery systems (Figs. 9 and 10), decreasing sheath sizes, offering a better coverage of the annulus (additional valve sizes), and facilitating sealing and positioning of the bioprosthesis. Technical advances are demonstrated on the successive generations of the balloon expandable and self-expandable transcatheter valves (Fig. 9).

The SAPIEN XT featured a lower profile delivery system, compatible with the new 18-20F e-Sheath designed to treat a broader population of patients and to reduce vascular complications. The valve consisted of an enhanced designed trileaflet bovine pericardial valve with a polyethylene terephlalate (PET) fabric cuff, sutured into a cobalt-chromium balloon-expandable stent with a modified geometry. Valves sizes were 23 mm, 26 mm and 29 mm. A 20 mm size was later available. Enhanced delivery systems were conceived for both transfemoral and mini-surgical approaches.

As evaluated in the SOURCE-XT registry (2688 patients in 99 European centers), the results confirmed important clinical benefits with a marked decrease of vascular complications and bleeding, and a decrease of all causes of mortality and cardiovascular mortality to 19.8% and 10.8% respectively at one year.^38^

The most important data came from the results of the randomized PARTNER 2 trial reported early this year.^39^ The trial enrolled 2,032 intermediate-risk patients at 57 centers, to undergo either TAVR or SAVR. At 2 years, non-inferiority of TAVR versus SAVR on rate of death or disabling stroke was demonstrated. Furthermore, in the transfemoral-access cohort, TAVR resulted in a significantly lower rate of death or disabling stroke than surgery. TAVR resulted in larger aortic-valve areas and lower rates of acute kidney injury, severe bleeding, and new-onset atrial fibrillation - whereas surgery resulted in fewer major vascular complications and less paravalvular aortic regurgitation. This led the FDA to extend approval of TAVR to intermediate risk patients.

Further progress came with the launch of the SAPIEN 3, the newest member of the SAPIEN family. The main improved features were a lower profile (compatible with 14-16F e-Sheath) allowing us to perform TAVR in about 90% of cases, an improved delivery system for more accurate positioning, and an external skirt to reduce paravalvular regurgitation. The SAPIEN 3 was approved in Europe in 2014 and in the US in 2015 for the treatment of high-risk and inoperable patients. Outcomes for high- and intermediate-risk patients treated with the SAPIEN 3 have been evaluated in the PARTNER II S3 trial,^40^ a nested registry of the PARTNER II Trial.

It reported 1-year follow-up in 1,077 intermediate risk patients implanted with SAPIEN 3 and compared outcomes using propensity score analysis, to the 747 patients treated with SAVR in the PARTNER 2A trial. For the primary endpoint of mortality, stroke, and moderate to severe aortic regurgitation, TAVR was superior to SAVR at one year (*p* < 0.001). The study showed the lowest rate of mortality, stroke and aortic regurgitation at 1 year of all SAPIEN trials and a superiority of TAVR over SAVR for these composite endpoints (*p* < 0.001). The conclusions suggested that TAVR might become the preferred treatment alternative in intermediate risk patients.

The Medtronic Evolut R is the new generation of the CoreValve self-expanding THV. The valve has been re-engineered to improve anatomic fit and sealing, to provide a more consistent radial force, to facilitate repositioning and retrieval, and reduce paravalvular leak. On a limited series of patients, the 30-day data showed a low rate of moderate to severe PVL and pacemaker implantation in comparison to previous Medtronic CoreValve series (3.4% and 12.4% respectively).^41^ This valve is currently approved in the United States for high- and extreme-risk patients with symptomatic severe AS.

The field of TAVR is constantly evolving. A number of next-generation devices, markedly different to existing devices, are in clinical evaluation and already CE accredited (Fig. 10). They incorporate features to reduce delivery catheter profile, facilitate positioning (repositionability), retrieval, and reduce paravalvular AR. However, it is too early to say whether these new bioprosthesis will represent the future of TAVR, but these advances create an active and stimulating competition. As examples, the LOTUS (Boston Scientific Marlbourough, MA, USA), comprises a nitinol frame with bovine pericardium valve released by an original mechanism offering optimal recapture, the DIRECT FLOW MEDICAL (Direct Flow medical, Lake Forest, CA, USA), comprises a rigid scaffold with a bovine pericardium valve and two inflatable aortic and ventricular rings, which almost eliminate paravalvular regurgitation.

## From trials to day-to-day practice: the growing place of TAVR as a breakthrough technology

In parallel to the advances in technologies, additional tools were developed regarding patient screening and procedures (new multimodality imaging technologies leaded by Multislice Computed Tomography), vascular complications (improved vascular closure devices), and stroke (embolic protection devices). Even the procedural “milieu” was modified with the development of a hybrid environment allowing integration, in the same setting, of interventional and surgical therapies. This testifies to the considerable impact of TAVR on the world of industry.

Thanks to these technological advancements, greater clinical experience, and the excellent results of post-market registries and evidence-based trials, TAVR has been brought to the fore as a treatment for AS and now appears in US and European guidelines. TAVR is indicated in patients with severe symptomatic AS who are not suitable for surgery, as assessed by a multidisciplinary heart team (Heart Valve Team) comprising cardiologists, cardiac surgeons, imaging specialists, anesthetists and other specialists including geriatricians.

TAVR should also be considered in high-risk patients who may still be candidates for surgery, but in whom a less invasive approach is favored, based on individual risk profile, including frailty. New guidelines in 2017 are expected to approve TAVI in intermediate risk patients. Another approved indication of TAVI is the treatment of failing surgical bioprosthetic heart valves (valve-in-valve). In this indication, TAVR is particularly appealing to achieve adequate valvular function for symptom relief without prolonged recovery. This indication is being evaluated in an ongoing global multicentre registry - Transcatheter Aortic Valve Implantation in Failed Bioprosthetic Surgical Valves.^42^ This new, less invasive therapeutic option for degenerated cardiac valve is pushing surgeons to increasingly select bioprosthetic instead of mechanical valves for primary valve replacement.

Subsequent to FDA approval, many centers were certified to apply TAVR in USA, currently nearly 500 centers, with around 26,000 patients included in the Society of Thoracic Surgery / American College of Cardiology Transcatheter Valve Therapy (TVT) registry.^43^ An equivalent number of centers are certified in Europe, with Germany being leader with 160 TAVR/million of inhabitants, followed by Switzerland, Austria and France.

**Figure 11.**
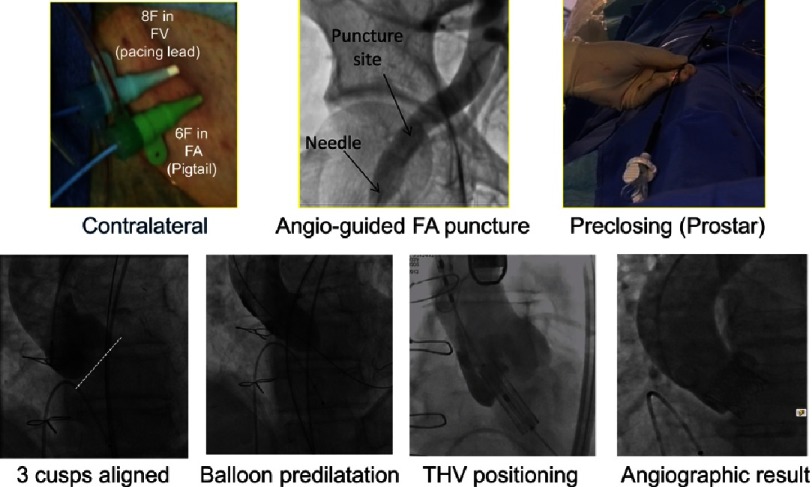
The different phases of transfemoral TAVI using the “minimalist” approach (SAPIEN 3 implantation).

The cost-effective “minimalist strategy” (Fig. 11) for transfemoral TAVR that we pioneered,^44,45^ plays an important role in the worldwide expansion of TAVR. It includes percutaneous transfemoral access, no general anaesthesia, no periprocedural transesophageal echocardiography, reduced operators in the room, and early discharge programs. This strategy can be applied in 90% of all TAVR patients, shows equivalent clinical outcomes compared to the standard transfemoral approaches, and is cost-effective.^46^

**Figure 12.**
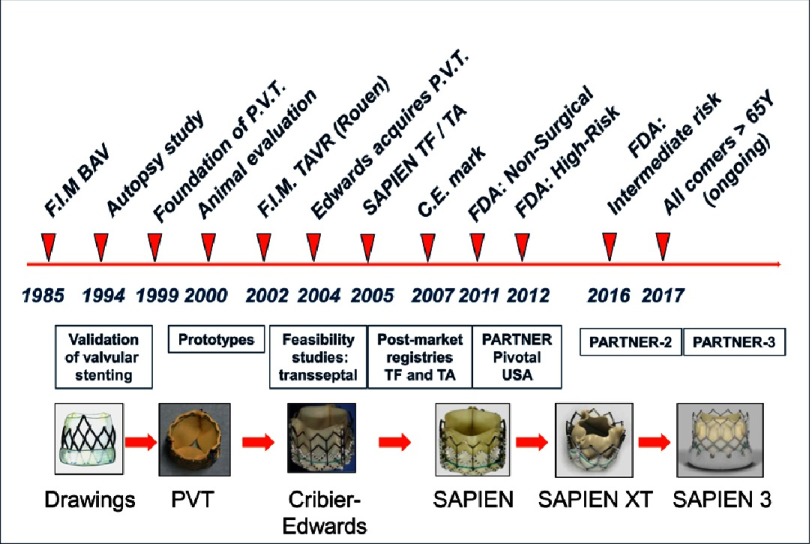
Development of the balloon expandable valve: an ongoing odyssey.

In the near future, TAVR will be extended to younger, lower-risk patients as reflected by the results of the PARTNER 2 and PARTNER 2 S3 studies. Using TAVR in “all comers” is already being evaluated. The Nordic Aortic Valve Intervention Trial (NOTION) first randomized almost 300 patients older than 70 years with severe aortic-valve stenosis but deemed low risk for surgery at three European centers. One-year results showed no significant differences in the composite rate of death from any cause, stroke, or MI (the primary outcome) between those undergoing TAVR and those undergoing SAVR.^47^ The ongoing PARTNER 3 trial started in 2016, with the goal of comparing TAVR and SAVR in all comers older than 65 years. A similar trial is ongoing with the Medtronic CoreValve. The results of these studies should have enormous consequences on the indications of TAVR in the future.

For the time being, the durability of THV compared to surgical heart valves remains unknown and has to be confirmed over the long-term. Our knowledge on long-term clinical follow-up is currently limited, but results are very encouraging. Normal valve function has been reported more than five years after TAVR^48^ and very few cases of failed THVs have been reported so far. As an anecdote, two of our patients have reached 10 years follow-up without any change in hemodynamics and no device deterioration.

## Conclusions

The development of TAVR has been a 20-year long inspiring and successful journey from concept to real world (Fig. 12). TAVR appears today a breakthrough technology, challenging the foundations of medical practice, enabling thousands of patients with severe AS to receive a life-saving effective alternative treatment to SAVR. This would not have been possible without the excellent and unequalled collaborative spirit between clinicians and engineers who have provided their expertise with the unique goal of making this procedure not just possible, but also safe and successful. We are not reaching the end of the story. The continuous translational work promises further technological innovations that will soon make TAVR simpler and safer. Within 10 years, it is likely that TAVR will become the default strategy for patients with symptomatic AS.
